# The Relationship Between Fears of Cancer Recurrence and Patient Gender: A Systematic Review and Meta-Analysis

**DOI:** 10.3389/fpsyg.2021.640866

**Published:** 2021-02-22

**Authors:** Chuan Pang, Gerry Humphris

**Affiliations:** ^1^Department of General Surgery, Chinese PLA General Hospital First Medical Center, Beijing, China; ^2^Division of Population and Behavioural Sciences, Medical School, University of St Andrews, St Andrews, United Kingdom

**Keywords:** cancer recurrence, fear, meta-analysis, gender, demographic characteristics

## Abstract

**Background:** A significant concern for patients treated for cancer is fear of cancer recurrence (FCR). Although a common experience, some patients report high levels of FCR that are difficult to manage and result in over vigilant checking and high use of health services. There has been speculation about the relationship of FCR with gender with mixed reports from several systematic reviews.

**Aims:** To determine the association of FCR with gender in previous reported studies and investigate the strength of this relationship with various moderators including year of publication, type of cancer and measurement attributes of self-reported FCR instruments.

**Methods:** A systematic review was conducted with searches of the literature from the MEDLINE, PubMed, Embase, and PsycINFO databases following PRISMA guidelines. All the included papers were divided into two groups, namely: “pure” that comprise only of patients with cancer types that both men and women can contract and “mixed” that report on patients with a variety of cancer types. The association between gender and FCR level was assessed by meta-analysis. A meta-regression was performed to investigate the moderating effects of factors including: the year of publication, cancer type, mean age of the sample and the length of the FCR scale measurement. This review was registered with PROSPERO, ID: CRD42020184812.

**Results:** Finally, 29 studies were included. The *N* size of pooled participants was 33,339. The meta-analysis showed females to have an overall higher level of FCR than males (ES = 0.30; 95% CI, 0.23, 0.36). The meta-regression of moderating or control variables found little, if any, systematic variation in effect-sizes.

**Conclusion:** This systematic review has clarified a potentially confused pattern of previous results in understanding the relationship between gender and FCR. Women report higher levels of FCR than men and this feature is one that clinicians and researchers can factor into their practice and future studies. The effect size is moderate, hence there is ample variation in FCR level, independent of gender, that requires further investigation.

## Introduction

Fear of cancer recurrence (FCR), or fear of progression (FoP) has been shown to be prevalent among cancer survivors, ranging from a normal reaction to a clinically significant level (Yang et al., 2017; Borreani et al., 2020). Through a rigorous consensus-based procedure in 2016, the latest commonly accepted definition of FCR is “fear, worry, or concern relating to the possibility that cancer will come back or progress” (Lebel et al., 2016). It is commonly reported to be the most significant concern of cancer survivors and the most frequent issue they want to discuss in consultations (Spencer et al., 1999; Lebel et al., 2007; Rogers et al., 2009; Ashing-Giwa and Lim, 2011). It has also been one of the most intensively studied areas of cancer-related health worries and unmet needs (Deimling et al., 2006b; Tsay et al., 2020). High-level FCR can lead to excessive checking behaviors and psychological distress, estimated to feature in 10% of cancer patients, as well as significant effects on associated mental health constructs such as depression and quality of life (QoL) (Hodges and Humphris, 2009; Tsay et al., 2020).

Researchers have been investigating factors associated with high FCR level. Demographic characteristics, such as gender, younger age, poorer education and lower income, may predict higher FCR level. Several studies have reported that females experience higher FCR than men (Wagner et al., 2018; Götze et al., 2019; Leclair et al., 2019), while others have not found any significant association between gender and FCR (Mullens et al., 2004; Steele et al., 2007; Jeon et al., 2019), and very few studies reported higher FCR in males (Yang et al., 2019; Luo et al., 2020). To date there has been no review exclusively focusing on the gender difference of FCR. Some comprehensive systematic reviews investigated factors that influence FCR level, including gender (Crist and Grunfeld, 2013; Koch et al., 2013; Simard et al., 2013). The results of these studies were somewhat contradictory. That is, they noted either that no results were provided to show a gender difference comparison or that there appeared to be little consensus, even when gender differences were explored. For instance, in Simard's review, only 4 studies reported significant association between genders and FCR while 12 other studies reported “no significance.” Hence the number of studies to examine a possible gender difference was relatively small, and none of these reviews were able to draw a definite conclusion about the role of gender.

Some cancers are gender-specific, such as ovarian, cervical, uterus and prostate cancers, which only females or males could contract. Breast cancer can be diagnosed in very few cases of men, however, in terms of its rareness it is usually regarded as a gender-specific cancer type as well. When assessing the factor of gender, many studies included both gender-specific cancer types and general cancer types, which both male and female can contract. This may produce biased gender-related results because gender-specific cancers only include patients of one gender, which may result in a mix of gender and cancer type factors and a biased gender distribution in a study sample. For example, more than half of the sample of Stephens' study (Stephens et al., 2016) are breast/uterus and prostate cancers. The former are all women, and the latter are all men. So, in this part of the sample (3,461 out of 6,099), different genders had different cancer types. It is hard to identify the exact effect level that either gender or cancer type is responsible for. This might explain for example why Simard and Savard's study found that women reported higher FCR, which included gender-specific cancer types such as breast and prostate cancers, but the association disappeared when cancer types were controlled (Simard and Savard, 2009). Moreover, gender-specific cancers may result in additional mental health related problems because these cancers are usually associated to the genital system, sexual characteristics, and hormonal differences (i.e., biological factors). It has been reported that some breast cancer patients may undergo significant mental health problems due to the negative psychological impact of the disease itself and the experience of the treatment process (Capuron et al., 2000; Ganz, 2001). Therefore, when analyzing the factor of gender, it is necessary to control cancer types and especially distinguish between gender-specific cancers and general cancers.

The reason to explore this potential gender difference with greater attention is that the clinician can use the gender classification as a potential reliable indicator of FCR. A similar remark has already been raised by Lim and Humphris in their review of FCR and patient age (Lim and Humphris, 2020). Hence, the clinician who is aware of the patient's gender (in addition to knowledge of the patient's age) may have the potential to predict somewhat the FCR in patients attending out-patient clinics. The present study therefore aims to conduct a systematic review of quantitative studies to investigate the association between gender and FCR level. Studies including gender-specific cancers and those only including cancer that can be contracted by both genders are analyzed, respectively, and compared. Furthermore, to understand the reported systematic variation of discrepancies between men and women in FCR level, we examined the possible effects of publication year, cancer type, FCR measure and mean age of the study sample.

## Methods

### Protocol

The present systematic review is registered on PROSPERO (https://www.crd.york.ac.uk/PROSPERO/; ID: CRD42020184812).

### Literature Collection

The relevant studies (published between 01 April 2000 and 01 May 2020) were identified by searching MEDLINE, PubMed, Embase, and PsycINFO databases, adhering to The Preferred Reporting Items for Systematic Reviews and Meta-Analyses (PRISMA) systematic review and meta-analysis guidelines (Shamseer et al., 2015). We also included any study known to the research team that had been submitted or in press in peer-review journals.

The key search terms were (“fear” [MESH] OR worry OR concern OR anxiety) AND (“neoplasm” [MESH] or cancer or carcinoma) AND (“recurrence” [MESH] OR “neoplasm recurrence” [MESH] OR progression OR return OR relapse OR remit) AND (gender OR male OR female OR men OR women).

### Inclusion/Exclusion Criteria

Papers selected for inclusion had to (a) be published in peer-reviewed journals between 01 April 2000 and 01 May 2020; (b) be written in English; (c) be quantitative studies and (d) report the association between patient gender and FCR or fear of progression (FoP) level in their results. Qualitative studies, dissertations, editorials, conference abstracts and commentaries were excluded. In addition, studies that reported fear of recurrence of nonneoplastic or noncancerous diseases were excluded.

### Data Extraction

After removing duplicate studies, the titles and abstracts of potential references were reviewed, and unsuitable ones were excluded. Then full texts were acquired and examined. The papers that completely fulfilled the inclusion criteria were conserved and recorded. Data extraction was conducted by CP and overviewed by GH. The following data were extracted for each study: (a) Authors' names, (b) year of publication, (c) sample size of the study, (d) mean age of the sample, (e) cancer types, (f) statistical data on gender and FCR/FoP association, (g) difference direction (males or females that have higher FCR/FoP), (h) FCR/FoP measure utilized (i) country of study, and (j) study design.

For papers incorporated more than one wave of valid data collection from different samples, each data collection was, respectively, extracted as independent studies in our review protocol. Where studies incorporated longitudinal waves of data collections from the same sample, we decided *a priori* to extract the association statistic from the baseline wave. The logic of selecting the first instance of patient assessment in a panel study was that it would likely have the largest sample size and hence favorable statistical power.

Based on the cancer types included in each of the study samples, all the included papers were divided into two groups. Group 1 (pure group) included studies that exclusively have cancers of one site or one system without gender-specific types. Hence the patients in this group will have contracted cancer such as head and neck cancer that affects both men and women. Whereas, group 2 (mixed group) included the studies with a number of cancer types including, or not including, gender-specific cancers, such as studies with mixed ovarian, prostate and other cancers. Although breast cancer can be contracted by men in rare cases, it is also regarded as a gender-specific type because the vast majority of the patients are women. Only one study in the mixed group does not have gender-specific cancers (Langeveld et al., 2004). Thus, in the pure group, we will be able to exclude the potential bias caused by gender-specific cancers or other various cancer types, while the studies with mixed cancer types were also analyzed so that it could be compared with the pure group to see if significant differences do exist. The cancer type will also be analyzed globally as a moderator in the following meta-regression to further investigate its possible effect on our results.

### Quality Assessment

The Joanna Briggs Institute (JBI) critical appraisal checklist for analytical cross-sectional studies was modified and applied to the cross-sectional, longitudinal, and RCT studies included in the present review (Aromataris et al., 2015; Lim and Humphris, 2020). The JBI checklist was an 8-item quality assessment tool. We excluded two items for our review which were not applicable: “was the exposure measured in a valid and reliable way” and “were objective, standard criteria used for measurement of the condition.” The assessment selections of “unclear” and “not applicable” were merged into one. The 6-item tool was manually applied to all 29 studies included in this review. The modified checklist is presented in the Supplementary Material.

### Statistical Analysis

#### Meta-Analysis

The meta-analysis of the two groups was conducted according to PRISMA guidelines to assess the association between gender and FCR in the included studies. The effect sizes were calculated using Comprehensive Meta-analysis routines (version 2.0). The raw correlation, odds ratio, *t*-test statistics or regression coefficients were obtained from the original published papers for conversion to an effect size in the analysis.

#### Heterogeneity

The heterogeneity estimates the variance among included studies, demonstrating the difference between the true effect size and the observed effect size. Measures of heterogeneity include *Q* value (random error), *T*^2^ (variance of effect sizes), *T* (standard deviation of effect sizes), and *I*^2^ (percentage heterogeneity) (Borenstein et al., 2009). We adopted a random effects model as the results tend to be more conservative and is considered more meaningful. That is, it calculates an effect size that can be referred to as a population estimate, as opposed to a fixed effects model that is more limited in its reference to the studies included in the review. In other words the random-effects model focuses not only on the differences in the effect-size in each study but also the sampling variability (chance).

#### Publication Bias

A conventional approach to publication bias was performed by plotting a “funnel plot” of the selected studies. Studies that reside outside pre-specified barriers or constraints can be identified. The number and patterns of these publications in occupying outside recognized contours would alert the review researcher to results that may be biased. The formal statistical tests, namely: Eggers and Beggs for reporting small study bias were performed. We also ran a procedure known as “one study removed” to assist with the detection of a single study that may distort the overall effect size estimation. The meta-analysis was rerun repeatedly and dropping in turn each study and replacing the previous omission. The purpose of this commonly utilized approach was to identify any major study that would influence unduly the final set of included studies.

#### Meta-Regression

Meta-regression was used to evaluate the association between one or more independent variables and effect size. It can be compared with multiple regression because similar statistical methods are used and it is possible to assess the relationship between the determined quality (or the moderator variable) and the effect size of each study (Thompson and Sharp, 1999; Higgins, 2011).

The analysis was performed adopting as the dependent variable the effect size for each study as displayed in the forest plot. With 29 studies therefore we have 29 effect sizes. We then include as independent variables the 4 moderator variables including the year of publication, cancer type, mean age of the sample and the length of the FCR scale measurement (single-item or multi-item).

The statistical results were produced by STATA15 software. The measures of heterogeneity were also generated, such as *T*^2^, *I*^2^, adjusted *R*^2^, and z value. The alpha level was set to the 0.05 (double-sided).

## Results

### Study Selection

**Figure 1:**
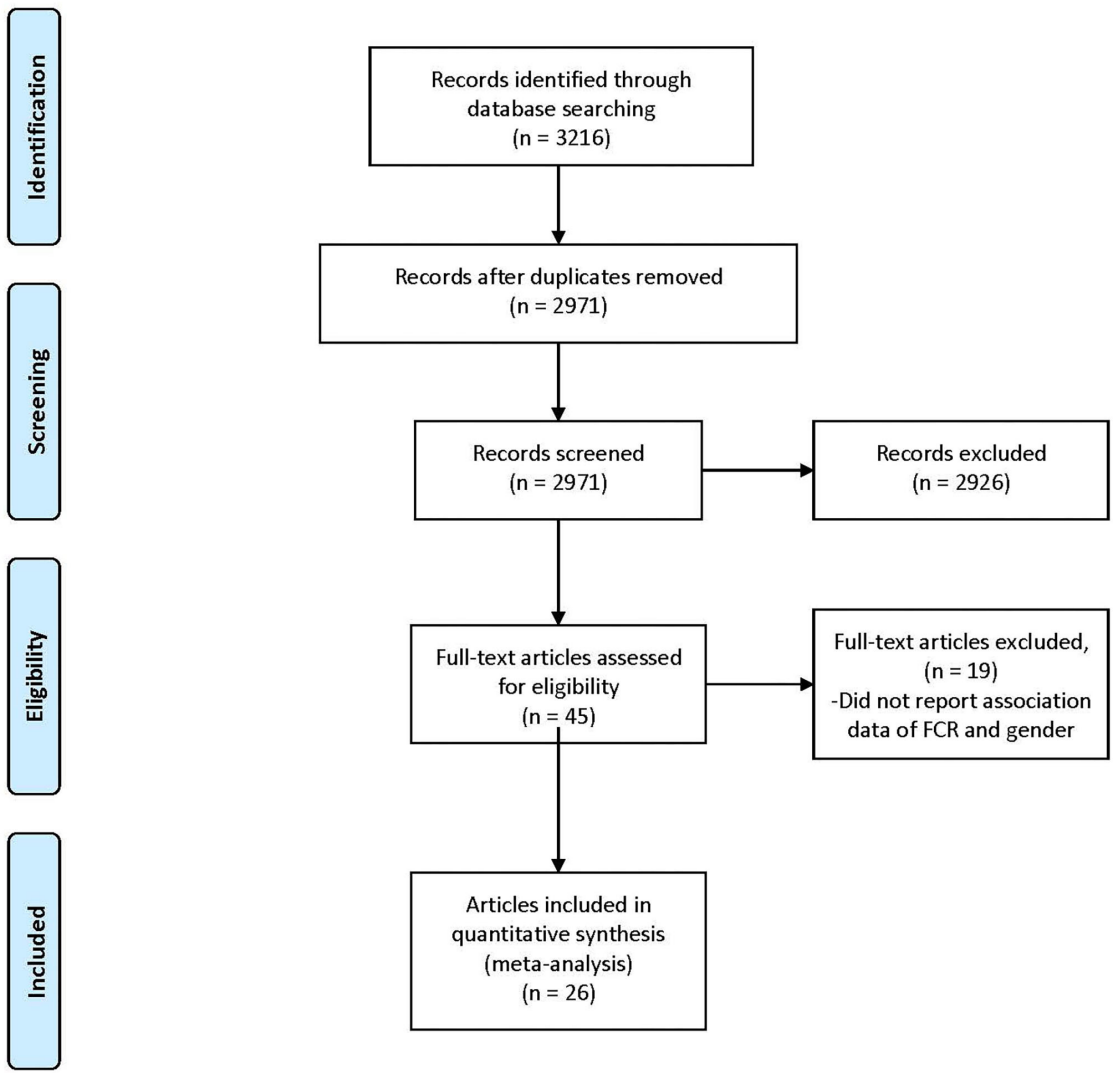
Preferred reporting items for systematic reviews and meta-analyses (PRISMA) diagram.

The search process is shown in Figure 1. In total, 3,216 references were identified from the four databases (EMBASE, MEDLINE, PubMed, and PsycINFO). After the duplicates were excluded, the remaining 2,971 titles and abstracts were scrutinized for relevance. Preliminary screening identified 45 papers to be relevant. The full texts were obtained, and the data regarding gender and FCR level were extracted. Finally, 26 papers were included. As described in the Data Extraction section, two included papers each incorporated two waves of data collection of different samples (Humphris et al., 2003; Deimling et al., 2006a). These four waves of data were extracted as four independent studies in the present review for analysis, entitled as Humphris^a,b^ and Deimling^a,b^ in Table 1. Additionally, one set of data was extracted from the Head & Neck 5000 study database, entitled as (Head Neck 5000 - NHS Foundation Trust, 2016^*d*^) and is being submitted. Hence the total number of studies for analysis is 29, out of which 15 were included in pure group and 14 were included in mixed group (Tables 1A,B).

**Table 1A:** Summary characteristics of the included studies in pure group.

**References**	**Sample size (N)**	**Mean age (SD)**	**Caner type**	**Main results**	**Higher FCR**	**FCR measurement**	**Country**	**Study design**
**Pure**								
Wagner et al. (2018)	136	54.3 (12.6)	Melanoma	Mean score (SD) Female = 32.2 (±8.4) Male = 28.5 (±8.0)	Female	FoP-Q-SF	Germany	Cross-sectional
Koch-Gallenkamp et al. (2016)	1,052	69	Colorectal Cancer	Mean score (SD) Female = 28.5 (9.6) Male = 25.6 (8.2)	Female	FoP-Q-SF	Germany	Cross-sectional
Sarkar et al. (2014)	239	50.4 (12.6)	Hematological Cancers	Multiple linear regression analysis b = 4.45	Female	FoP-Q-SF	Germany	Prospective clinical trial
Mirosevic et al. (2019)	216	62.3 (9.7)	Head and Neck Cancer	Age-adjusted univariate regressions Beta = 0.337 *t* = 0.502 *P* = 0.62	NS	CWS	the Netherlands	Cross-sectional
Humphris et al. (2003)^a^	87	58.3 (11.3)	Orofacial Cancer	Recurrence concern frequency; *n* (%) Male 50 (82%) Female 22 (85%)	NS	CWS	UK	Prospective (Sample 1)
Humphris et al. (2003)^b^	50	58.3 (11.4)	Orofacial Cancer	Recurrence concern frequency; *n* (%) Male 22 (63%) Female 10(67%)	NS	CWS	UK	Cross-sectional with follow up (Sample 2)
Borreani et al. (2020)	75	N/A	Hematological Cancer	Multiple linear regression *B* = 0.416 SE = 0.153 β = 0.306 *t* = 2.724	Female	FCRI	Italy	Cross-sectional
Fisher et al. (2016)	10969	≥65 6,772 <65 4,197	Colorectal Cancer	Binary logistic regressions; Male = 1 OR (95% CI) 1.59 (1.48, 1.72)	Female	Single question	UK	Cross-sectional
Deimling et al. (2006a)^a^	96	N/A	Colorectal Cancer	Mean (SD) Female 10.9 (3.2) Male 11.7 (3.1)	Female	4 items	USA	Cross-sectional
Mullens et al. (2004)	41	56.1	Colorectal Cancer	Women 2.08 (0.88) Men 2.07 (0.64)	NS	Single question	USA	Cross-sectional
Steele et al. (2007)	91	≤ 60 years 32 61–70 years 39 >70 years 20	Colorectal Cancer	Chi-square test *p* = 0.52	NS	Single question	UK	Cross-sectional
Rogers et al. (2010)	123	<55 years 32 55–64 years 45 65+ years 46	Head and Neck Cancer	Chi-square *p* = 0.86	NS	PCI	UK	Clinical cohort trial
Erim et al. (2013)	70	58.1 (15.5)	Malignant Melanoma	Multiple linear regression Z = −2.447; *p* = 0.014	Female	FoP-Q	Germany	Cross-sectional
Humphris et al. (2018)	53	67	Colorectal	Women 9.000 (3.406) Men 8.563 (4.071)	Female	FCR4	UK	Cross-sectional
Head Neck 5000 - NHS Foundation Trust (2016)	3195	<50 years 13.6% ≥50 years 86.4%	Head and Neck Cancer	Women 11.86 (4.05) Men 10.62 (3.90)	Female	FCR4	UK	Prospective longitudinal

*The ^a,b^ mean to differentiate them as described in Results-Study Selection*.

**Table 1B:** Summary characteristics of the included studies in mixed group.

**References**	**Sample size (*N*)**	**Mean age (SD)**	**Caner type**	**Main results**	**Higher FCR**	**FCR measurement**	**Country**	**Study design**
**Mixed**								
Götze et al. (2019)	1,002	66.7 (10.5)	Mixed	Mean (SD) Male22.1 (8.4) Female 27.4 (9.5) effect size *d* = 0.593	Female	FoP-Q-SF	Germany	Cross-sectional
van de Wal et al. (2016)	2,615	63.6 (12.9)	Mixed	Male 2.64 Female 2.84 (1.00) *t* = −4.898 effect size d = 0.19	Female	IOC-HWS	The Netherlands	Cross-sectional
Stephens et al. (2016)	6,099	<65 *n* = 2,595 65 *n* = 3,499	Mixed	Mean(SD) Male 0.85 (1.10) Female 1.26 (1.28)	Female	CPILS	USA	Cross-sectional
Matthews (2003)	123	54.96	Mixed	Analysis of covariance, Mean(SD) Male = 5.64 (SD 3.44) Female = 5.69 (SD 3.84) *F* = 0.01 df = 1, 121	Female	QOL-F/CS	USA	Cross-sectional
Baker et al. (2005)	752	18–54 years 50% >54 years 50%	Mixed	Chi-square tests OR (95% CI) 1.5 (1.23–1.75)	Female	Single item	USA	Prospective longitudinal
Simard and Savard (2009)	600	Lung 62 (1.5) Breast 59 (0.6) Colorectal 61.6 (1.3) Prostate 69.1 (0.5)	Mixed	Correlation *r* = 0.31	Female	FRQ	Canada	Cross-sectional
Mellon et al. (2007)	207	65 (6.2)	Mixed	Point biserial correlations *r* = 0.04	NS	FRQ	USA	Cross-sectional
Langeveld et al. (2004)	400	24 (4.9)	Mixed	Mean (SD) Male 7.3 (2.7) Female 8.2 (3.0) effect sizes *d*= 0.31 *p* < 0.05 *t*-test	Female	Single item	Netherlands	Cross-sectional
Deimling et al. (2006a)^b^	321	72.3(7.5)	Mixed	Regression 1 = Female Item 1–4: *r* = −0.07, −0.02, −0.02, −0.02	Female	Four items	USA	Cross-sectional
Gemmill et al. (2010)	307	74 (8.7)	Mixed	Mean score Female 7.0 Male 7.9 *p* = 0.02	Female	HRQOL	USA	Cross-sectional
Hinz et al. (2014)	2059	62.4	Mixed	Two-way ANOVAs *d* (effect size) = 0.52	Female	FoP-Q-SF	Germany	Prospective
Mikkelsen et al. (2009)	340	59.5 (11.5)	Mixed	Positive answers number, *N* (%) Male 51 (40.8) Female 125 (58.1)	NS	Single question	Denmark	Cross-sectional
Luo et al. (2020)	996	48.04 (11.71)	Mixed	Multivariable logistic regression Female Exp (*B*) = 1.292 *P* = 0.348	Male (NS)	FoP-Q-SF	China	Cross-sectional
Yang et al. (2019)	1025	<35 years 14.2% 35–60 years 65.9% >60 years 19.9%	Mixed	*t* (df) = 1.13 (1,023)	Male (NS)	FCR7	China	Cross-sectional

*Abbreviations of FCR measurements: FoP-Q, Fear of Progression Questionnaire; FoP-Q-SF, Fear of Progression Questionnaire-Short Form; CWS, Cancer Worry Scale; FCRI, Fear of Cancer Recurrence Inventory; PCI, 45-item Patient Concerns Inventory; FCR4 or FCR7, 4 or 7 item version of the Fears of Cancer Recurrence Scale; IOC-HWS, The Health Worries subscale of the Impact of Cancer scale; CPILS, Cancer Problems in Living Scale; QOL-F/CS, Quality of Life–Family/Cancer Survivor; FRQ, Fear of Recurrence Questionnaire; HRQOL, Health-related quality of life*.

### Quality Assessment

Studies were all satisfactory as they were rated positively in half or more of the six criteria (Tables 2A,B).

**Table 2A:** Quality assessment by modified Joanna Briggs tool on the included studies in pure group.

**References**	**Item 1 inclusion criteria**	**Item 2 subject details**	**Item 3 confounds**	**Item 4 strategies**	**Item 5 measure-ments**	**Item 6 statistics**	**Overall score (6 max)**
**Pure group**							
Humphris et al. (2003)^a^							5
Humphris et al. (2003)^b^							5
Mullens et al. (2004)							5
Deimling et al. (2006b)^b^							6
Steele et al. (2007)							3
Erim et al. (2013)							5
Sarkar et al. (2014)							6
Koch-Gallenkamp et al. (2016)							6
Fisher et al. (2016)							4
Wagner et al. (2018)							6
Humphris et al. (2018)							4
Mirosevic et al. (2019)							6
Borreani et al. (2020)							6
Rogers et al. (2010)							4
Head Neck 5000 - NHS Foundation Trust (2016)							6

Green = “Yes”; Red = “No”; Yellow = “Unclear/Not applicable.”

*The ^a,b^ mean to differentiate them as described in Results-Study Selection*.

**Table 2B:** Quality assessment by modified Joanna Briggs tool on the included studies in mixed group.

**References**	**Item 1 inclusion criteria**	**Item 2 subject details**	**Item 3 confounds**	**Item 4 strategies**	**Item 5 measure-ments**	**Item 6 statistics**	**Overall score (6 max)**
**Mixed group**							
Matthews (2003)							6
Langeveld et al. (2004)							5
Baker et al. (2005)							5
Deimling et al. (2006b)^a^							6
Mellon et al. (2007)							6
Simard and Savard (2009)							4
Mikkelsen et al. (2009)							5
Gemmill et al. (2010)							4
Hinz et al. (2014)							4
van de Wal et al. (2016)							6
Stephens et al. (2016)							6
Götze et al. (2019)							6
Luo et al. (2020)							6
Yang et al. (2019)							6

Green = “Yes”; Red = “No”; Yellow = “Unclear/Not applicable.”

### Overall Effect

**Figure 2:**
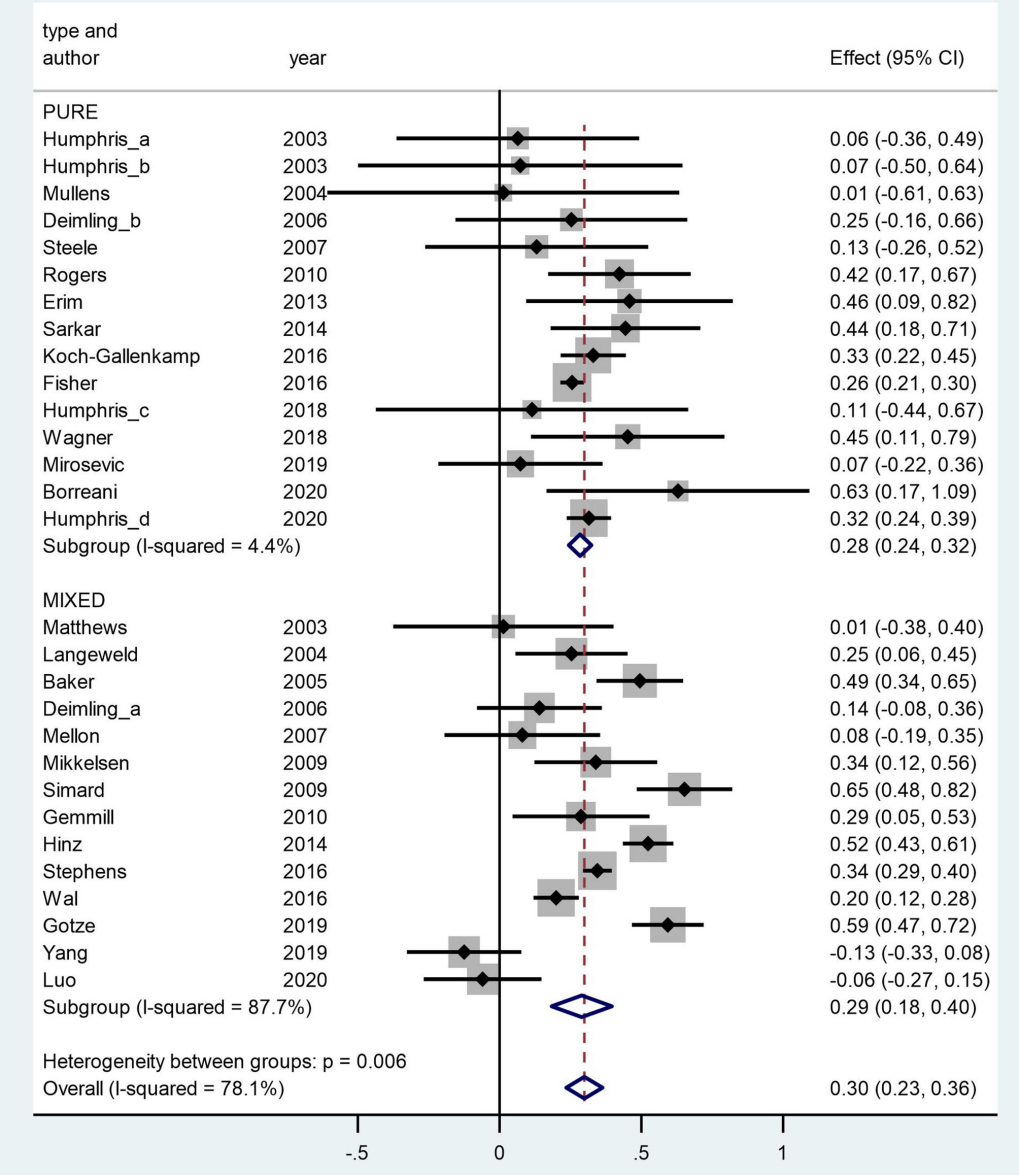
Forest plot of fears of cancer recurrence (FCR) and gender in a random-effects model. Weights are from random-effects model.

The N size of pooled participants was 33,339, 16,493 in the pure group and 16,846 in the mixed group. Two thirds of the included studies (66% i.e., 19 out of 29) reported that females have significantly higher FCR level. Two studies reported a higher average mean FCR score for males. These were conducted with separate samples from the Guangzhou Hospital Region (Yang et al., 2019; Luo et al., 2020). The forest plot (Figure 2) shows the effect sizes of the pure group and mixed group are, respectively, 0.28 (95% confidence interval: 0.24–0.32) and 0.29 (95% confidence interval: 0.18–0.40), with an overall effect size of 0.30 (95% CI: 0.23–0.36). The tests for the overall, pure, and mixed group effect size are, respectively, z = 9.16 (*p* < 0.001), z = 13.90 (*p* < 0.001), z = 5.34 (*P* < 0.001). With the “one study removed” analysis it was found that the minimum overall effect size reported was 0.284 demonstrating the very limited effect of any single study to influence the overall results. A classic Fail-safe *N* calculation resulted in 2,750+ studies would need to be found to bring the effect size to a not significant value (*p* > 0.05).

### Heterogeneity

The overall *Q* value for heterogeneity is 127.75 (df = 27, *p* < 0.001), *I*^2^ is 78%, and Tau^2^ is 0.034. The *Q* values of the pure group and mixed group are 14.65 (df = 14, *p* = 0.403) and 105.40 (df = 13, *p* < 0.001), respectively. The difference between the two groups is significant (*p* = 0.006).

### Publication Bias

**Figure 3:**
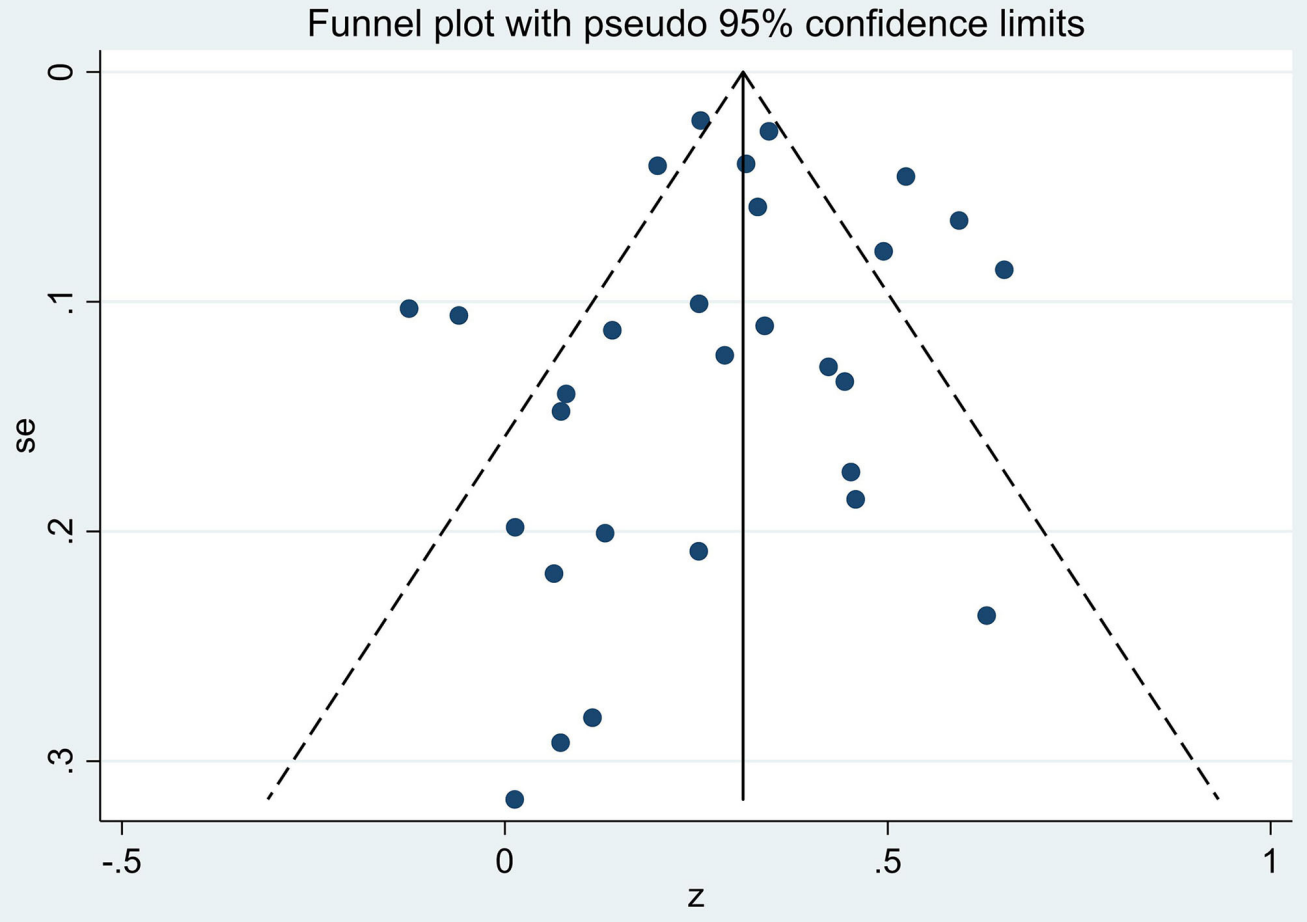
Funnel plot of the four covariates: year of publication, cancer type, age of sample, and FCR measurement on gender by fears of cancer recurrence (FCR) association effect sizes (z).

The Egger and Begg tests found no consistent evidence of reporting bias (z = −0.07, *p* = 0.94 and z = −0.99, *p* = 0.32, respectively). Likewise, the funnel plots showed little evidence of consistent bias (Figure 3) as shown by an approximate symmetric pattern on either side of the 95% CI boundary.

### Meta-Regression

A meta-regression was performed, including four moderators of the publication year (range = 2,003–2,020), cancer type (0 = pure or 1 = mixed), mean age (in years) of the sample (range = 24–74) and the length of the FCR measurement (0 = single-item or 1 = multi-item) (Table 3). No statistically significant effects of these moderators were found (all *p* levels > 0.4). In addition, we investigated the potential difference between single cancer type, such as the head and neck cancer, and the other cancers but again no reliable effect was shown (z = −0.26, *p* = 0.79).

**Table 3:** Meta-regression of effect sizes by four covariates: year of publication, cancer type, age of sample, and FCR measurement on gender by fears of cancer recurrence (FCR).

**Moderators**	**B coef**	**SE**	***t***	***P***	**95% conf. interval**	
Year of publication	0.000	0.008	0.02	0.983	−0.016	0.017
Cancer type	0.005	0.088	0.05	0.957	−0.176	0.185
Age of sample	0.003	0.004	0.75	0.459	−0.006	0.012
FCR Item length	0.047	0.105	0.45	0.660	−0.170	0.264

## Discussion

The present study is the first systematic review and meta-analysis to investigate the gender difference of FCR specifically. It demonstrated a significant association between gender and FCR level, with a moderate overall effect size of 0.30 in a pooled sample of 33,339 from 29 studies, and the two groups “pure” and “mixed” reported consistent effect sizes of 0.28 and 0.29, indicating females with greater FCR levels. Only two studies from Guangzhou, China reported slightly higher FCR in males but the differences are not statistically significant (Yang et al., 2019; Luo et al., 2020). The overall effect size value stays stable with the removal of any single study. The quality assessment assured a good methodological standard for all the included studies, and no consistent bias was found.

Previously, it seems an apparent consensus among clinicians that a gender difference of FCR exists. However, there are many studies demonstrating no significant association between gender and FCR level. Some previous comprehensive systematic reviews to investigate possible determinants of FCR involved gender in their analysis (Crist and Grunfeld, 2013; Koch et al., 2013; Simard et al., 2013). However, given that gender was not the focus of their analysis, its role was not discussed in detail. The result of the present review has clarified the potentially confused pattern of previous results in understanding the relationship between gender and FCR. That is, women report higher levels of FCR than men, and this feature is one that clinicians and researchers can factor into their practice and future studies.

### Possible Reasons for FCR Gender Differences

An explanation of the gender difference of FCR is likely to be multifactorial. The etiology of FCR development is complex and is poorly understood. The basis of how gender might influence FCR can be speculated upon from various sources. In general, gender differences are common and well-described in mental and psychological conditions among common people as well as cancer patients, and negative conditions are more prevalent in women than men (Faravelli et al., 2013; Salk et al., 2017; Aminisani et al., 2021). A recent study demonstrated that the frequency of psychological distress is especially high among women with colorectal cancers (Aminisani et al., 2021). Another study reported that female sarcoma patients have lower health-related quality of life (HRQoL) (Eichler et al., 2020). Specifically, first, gender differences of social and psychological indicators are associated to factors of income (Reiss, 2013), exposure to violence (Koss et al., 1995) and the division of labor by gender (Wood and Eagly, 2012). These social factors also apply to cancer patients, and women cancer patients usually need more social and mental support (Ozbayir et al., 2019). These have been explained as largely stemming from the gender inequality against women within society but vary widely on a cross-nation level (Salk et al., 2017). Second, women cancer patients are more inclined to express their problems and seek for help, while men patients may abstain from expressing fear or worry when suffering a negative condition because of feelings of shame (Clover et al., 2015; Anuk et al., 2019). This implies that women may tend to report higher FCR level.

### The Comparison Between Groups and the Heterogeneity

Many studies with mixed cancer types included gender-specific cancers when assessing the effect of gender. This may have potential impact on the gender distribution of study samples and gender-related analysis, and lead to bias of study results. Given this concern, we divided all the included studies into “pure” and “mixed” groups. The cancer type seems to have a very limited effect, and less than expected on effect sizes of the two groups, resulting in almost identical values (0.28 vs. 0.29). However, the heterogeneity of the mixed group is much greater than the pure group, leading the overall heterogeneity to a similarly high level. This was to be expected because studies of the mixed group have more diverse samples with various types of cancer including gender-specific cancers and in addition the authors adopted more complex data analytical approaches. They also used, on inspection, a more variable set of FCR measurement tools. Most of the studies of the pure group used standardized questionnaires such as the Fear of Progression Questionnaire (FoP-Q) or the 4 item version of the Fears of Cancer Recurrence Scale (FCR4), while in the mixed group, almost all the studies used study specific FCR measures ranging from a self-defined single question, 4-item scale, to subscales extracted from various other questionnaires such as the Cancer Problems in Living Scale (CPILS), the Health Worries subscale of the Impact of Cancer scale (IOC-HWS) or Health-related Quality of Life (HRQOL). The exception to this rule were three studies that consistently used the FoP-Q short form (Hinz et al., 2014; Götze et al., 2019; Luo et al., 2020). This possible complication, may reflect itself in the observed level of heterogeneity of the mixed group as well as the overall statistics. We consider this may not influence our conclusion substantially because the heterogeneity of the pure group stays at a very low level, and the pure group possessed an almost identical effect size as the mixed group and subsequently the overall result.

### Moderator Variables

We performed a meta-regression including four moderator variables of: cancer type, publication year, mean age of the sample and single- or multi-item FCR measurement, because these factors have potential effects on FCR level. FCR level differs among patients with different cancer types (Crist and Grunfeld, 2013; Simard et al., 2013), which was also discussed in the gender-specific cancer relevant section in this review. In a meta-analysis about the relationship between age and FCR, the authors found the more recent the study, the strength of the effect size decreases (Lim and Humphris, 2020). Several studies demonstrated a relationship between patient age and FCR. The older the cancer patients, the lower the reported FCR level (Simard et al., 2013; Lim and Humphris, 2020). Furthermore, given that there is no consensus on the measurement tool of FCR, the methods in literatures vary from the 43-item Fear of Progression Questionnaire (FoP-Q) (Erim et al., 2013) to a single-item question like “I have fear about my cancer coming back” (Fisher et al., 2016), which generate different FCR results. However, no significant result of the moderator variables was found in the present study, demonstrating they might have less effect than expectation. But in consideration of the previous positive evidence mentioned above, these factors are still important in future studies to be explored further.

### Clinical Implications

In clinical communication, clinicians play a crucial role and often occupy an important position from the patient's perspective. It has been recommended that clinicians should pay attention to the discussion of FCR with cancer patients. There are studies showing that non-mental health trained clinicians can provide effective interventions for FCR (Liu et al., 2019). But this is still challenging because non-mental health trained oncologists often have difficulties in deciding which patients need such assistance. There are tools developed to assist clinicians to be aware of cancer patients' individual needs, such as the Patient Concerns Inventory (PCI), a checklist where patients can select the items they want to discuss at a clinic, including the fear of cancer recurrence (Rogers et al., 2018). However, it may be of benefit if the more easily obtained demographic characteristics, such as gender and age, can be used as easily available indicators to help clinicians to focus on the discussion of FCR with patients at high risk. The result of this review fits our prediction and supports clinicians who consider gender as a useful indicator of FCR level. It might help clinicians to focus discussion of FCR with patients attending follow-up clinics when combine their gender with other easily obtained demographic characteristics, and help improve the efficiency of doctor-patient communication.

### Strengths and Limitations

This review has a number of strengths including the systematic search for all quality studies that reported the relationship between fears of recurrence or progression with gender. In addition, this is the first study to utilize meta-analytical and meta-regression methodology to assist interpretation of a substantial pooled sample of reported investigations. However, this systematic review and meta-analysis has several limitations. First, to completely avoid the potential bias from gender-specific cancers, only papers about both-gender cancers should be included. However, only sixteen studies have met this criterion, which provides some evidence but not extensive. Therefore, we included gender-specific cancers and divided these studies into two groups (“pure” and “mixed”) as outlined already with the additional analysis to study the effect of cancer types, that showed no statistical significance. Second, this review excluded papers not written in English, which may reduce its generalisabilty to some extent.

### Future Directions

In the literatures on FCR so far, gender has been usually regarded as a “not-so-important” factor out of various demographic characteristics. The effect of gender should be investigated with greater consideration and authors in the FCR field are encouraged to report FCR levels by gender. When attempting to assess the factor of gender, cancer types that only a single gender can contract should be considered carefully in their interpretation when intending to investigate gender FCR differences. An argument can be voiced to exclude such cancer types in assessing gender and FCR association to avoid possible confusion. In terms of the high-level heterogeneity observed in the mixed group of this review, while the mixed group of studies may have included a wider selection of patient samples the varied measurements employed indicated that an international consensus on the FCR measurement may be helpful to reduce the number of measures and possibly strengthen consistency. In addition, authors of future studies are commended to include in their manuscript results a breakdown or association statistic of gender and FCR to assist any review update and strengthen our understanding of this key relationship.

## Conclusion

In this systematic review of studies over the past 20 years, the previous confusing pattern of outcomes reported in the literature of the relationship between gender and FCR was clarified. Women report higher levels of FCR than men, a finding that clinicians and researchers can factor into their practice and future research with care to avoid possible stereo-typing. With only a moderate effect size, indicating that the level of FCR was somewhat variable, independent of gender, demonstrates that further study is required.

## Data Availability Statement

The original contributions presented in the study are included in the article/Supplementary Material, further inquiries can be directed to the corresponding author/s.

## Author Contributions

GH conceived the study, analyzed the raw data, and reviewed and edited the original draft. CP collected the papers, extracted the raw data and wrote the original draft. The whole study process was overviewed by GH and discussed regularly by GH and CP. All authors contributed to the article and approved the submitted version.

## Conflict of Interest

The authors declare that the research was conducted in the absence of any commercial or financial relationships that could be construed as a potential conflict of interest.
