# Supplementary material for: The Relationship Between Fears of Cancer Recurrence and Patient Gender: A Systematic Review and Meta-Analysis
Source: Front Psychol. 2021 Feb 22;12:640866. doi: 10.3389/fpsyg.2021.640866 (PMC7937637; doi:10.3389/fpsyg.2021.640866)
Supplement: Supplementary file 1 [file Presentation_1.PDF]

Modified items of Joanna Briggs Quality Assessment Tool

1. Were the criteria for inclusion in the sample clearly defined?

2. Were the study subjects and the setting described in detail?

3. Were confounding factors identified?

4. Were strategies to deal with confounding factors stated?

5. Were the outcomes measured in a valid and reliable way?

6. Was appropriate statistical analysis used?

(Scored as Yes: green; No: Red; Yellow: unclear/not applicable)
